# Microfluidic Light-Scattering Imaging Coupled with Deep Learning for Label-Free Single-Cell Classification of Lymphoma Cells

**DOI:** 10.3390/bios16090500

**Published:** 2026-09-07

**Authors:** Linyan Xie, Mengfei Wang, Xijia Luo, Shuoxian Xia, Qiongqiong Ren, Xuezhi Zhou

**Affiliations:** 1 School of Mathematical Medicine and School of Medical Engineering, Henan Medical University, Xinxiang 453003, China; 50240205007@stu.xxmu.edu.cn (M.W.); 50250205011@stu.xxmu.edu.cn (X.L.); 151036@xxmu.edu.cn (Q.R.); 2 Xinxiang Key Laboratory of Neurobiosensor, Xinxiang 453003, China; 3 Henan Engineering Technology Research Center of Neural Sensing and Control, Xinxiang 453003, China; 4 School of General Practice, Henan Medical University, Xinxiang 453003, China; 20241500604@stu.xxmu.edu.cn

**Keywords:** microfluidic, light-scattering imaging, deep learning, label-free, single-cell, lymphoma, cell classification

## Abstract

Accurate classification of lymphoma cell subtypes is essential for disease diagnosis and therapeutic decision-making, yet conventional approaches often rely on fluorescence labeling, labor-intensive sample preparation, and specialized instrumentation, limiting their applicability for rapid, label-free single-cell analysis. Here, we present an AI-assisted microfluidic light-scattering imaging platform for label-free classification of lymphoma cells. The platform integrates hydrodynamic focusing within a microfluidic chip, continuous acquisition of two-dimensional (2D) light-scattering patterns, automated image preprocessing, and transfer learning based on a pretrained ResNet50 network for intelligent optical feature extraction and classification. Human B lymphoma (Daudi) and T lymphoblastic lymphoma (SUP-T1) cells were used to evaluate the proposed framework. The optical imaging system was first validated using standard microspheres, demonstrating reliable acquisition of light-scattering patterns under continuous-flow conditions. A dataset comprising 800 single-cell scattering patterns was subsequently established and evaluated using stratified five-fold cross-validation. The proposed framework achieved an average classification accuracy of 94.75% with an average area under the receiver operating characteristic (ROC) curve of 0.986. By integrating microfluidic optical biosensing with deep learning, this work enables automated interpretation of intrinsic optical scattering signatures and provides a promising AI-enabled strategy for rapid, label-free lymphoma screening and intelligent healthcare applications.

## 1. Introduction

Lymphoma comprises a heterogeneous group of hematological malignancies originating from the abnormal proliferation and differentiation of lymphocytes. Owing to its substantial biological heterogeneity, accurate identification of lymphoma cell types is essential for early diagnosis, disease stratification, therapeutic decision-making, and prognosis evaluation [1,2,3,4,5]. Current clinical diagnosis primarily relies on histopathological examination [6,7], immunohistochemistry [8], flow cytometry [9], and medical imaging [10]. Although these techniques provide high diagnostic accuracy, they generally require labor-intensive sample preparation, fluorescence labeling, expensive instrumentation, and experienced personnel, making them less suitable for rapid, label-free, and high-throughput single-cell analysis [11,12,13,14]. Therefore, developing optical biosensing technologies capable of rapidly characterizing individual lymphoma cells without exogenous labeling has become an important objective in intelligent healthcare diagnostics.

The development of novel materials and label-free cell identification technologies provides technical support for cancer diagnosis and cellular phenotypic analysis, holding great application potential [15,16]. Among emerging label-free optical sensing technologies, vibrational spectroscopic techniques, including Raman and infrared spectroscopy, have demonstrated considerable potential for cancer diagnosis by exploiting intrinsic molecular fingerprints without exogenous labels [17,18]. In addition to spectroscopic approaches, light-scattering imaging has recently attracted increasing attention as a powerful label-free optical modality, because the scattered optical signals inherently encode the morphological characteristics, intracellular structures, and refractive-index distributions of biological cells [19,20,21]. Unlike fluorescence-based approaches, light-scattering imaging interrogates intrinsic optical properties without introducing external contrast agents, thereby minimizing sample perturbation and simplifying experimental procedures [22]. Meanwhile, microfluidic technology enables precise manipulation of individual cells within microscale channels through hydrodynamic focusing, providing stable cell alignment, reduced sample consumption, and continuous sample transport [23,24,25,26,27,28]. The integration of microfluidics with optical light-scattering imaging therefore offers an attractive biosensing platform for rapid and high-throughput single-cell characterization under continuous-flow conditions [29].

Despite these advantages, extracting diagnostically meaningful information from light-scattering patterns remains a significant challenge. Optical scattering patterns contain highly complex spatial intensity distributions that are influenced by cellular morphology, internal microstructures, and refractive index variations [30]. Previous studies have shown that optical spectroscopic approaches integrated with machine learning can accurately distinguish different cancer subtypes by learning intrinsic optical signatures, demonstrating the feasibility of AI-assisted optical diagnosis [17]. However, conventional analytical approaches typically depend on handcrafted feature extraction followed by traditional machine learning algorithms. Such methods often require extensive expert knowledge, are sensitive to imaging variations, and may fail to capture the nonlinear and high-dimensional characteristics embedded within scattering patterns [31,32,33]. Moreover, studies specifically focusing on continuous-flow light-scattering imaging for label-free lymphoma cell classification remain limited.

Recent advances in artificial intelligence, particularly deep learning, have fundamentally transformed biomedical image analysis by enabling automatic extraction of hierarchical feature representations directly from raw image data. Compared with conventional feature-engineering approaches, convolutional neural networks can simultaneously learn complex spatial features and optimize classification models in an end-to-end manner, thereby improving robustness and analytical efficiency [34,35]. Furthermore, recent studies have demonstrated its capability to improve image quality through denoising, aberration correction, isotropic resolution restoration, and automated feature extraction, substantially enhancing the analytical performance of optical imaging systems [36]. More importantly, deep learning has evolved beyond conventional image classification and has become an enabling technology for intelligent optical biosensing and computational microscopy [37,38,39,40]. The integration of deep learning with microfluidic light-scattering imaging therefore represents a promising strategy to overcome the limitations of traditional analysis methods and achieve accurate, scalable, and automated label-free single-cell cancer diagnosis [41,42,43,44,45].

In this study, we developed an AI-assisted microfluidic light-scattering imaging platform for the label-free classification of lymphoma cells at the single-cell level. The proposed platform integrates hydrodynamic focusing within a microfluidic chip, continuous acquisition of 2D light-scattering patterns, automated image preprocessing, and transfer learning based on a pretrained ResNet50 network to enable intelligent feature extraction and classification. Compared with existing research findings, our platform collects light-scattering signals within a specific range, largely preserving the complete scattering signals of cells while reducing interfering information, and enabling dynamic measurement, automatic extraction of image features and classification [43,46]. Human B lymphoma (Daudi) and T lymphoblastic lymphoma (SUP-T1) cells were employed to validate the proposed framework. Experimental results demonstrated that the platform enables stable acquisition of high-quality light-scattering patterns under continuous-flow conditions and achieves accurate label-free discrimination of individual lymphoma cells. By integrating microfluidic optical biosensing with deep learning, the proposed framework enables automated interpretation of intrinsic optical scattering signatures for intelligent single-cell analysis. This work provides a promising AI-enabled optical biosensing strategy for rapid, label-free lymphoma screening and highlights the potential of computational optical sensing technologies for future intelligent healthcare applications.

## 2. Materials and Methods

### 2.1. Microsphere and Cell Samples

To validate the performance of the optical light-scattering imaging system prior to biological experiments, standard silica (SiO_2_) microspheres (4 μm in nominal diameter) and polystyrene (PS) microspheres (6 μm in nominal diameter) (Zhongke Leiming Technology Co., Ltd., Beijing, China) were employed as reference samples. For each microsphere type, 1 μL of the stock suspension was diluted in 3 mL of deionized water to obtain a homogeneous suspension with a final concentration of approximately 3.0 × 10^3^ particles mL^−1^. The diluted suspensions were used for liquid-phase optical measurements to acquire 2D light-scattering patterns of individual microspheres. Immediately before each experiment, the suspensions were gently vortexed to ensure a uniform particle distribution.

Human B lymphoma Daudi cells (National Collection of Authenticated Cell Cultures, Shanghai, China) and human T lymphoblastic lymphoma SUP-T1 cells (Shanghai Qingqi Biotechnology, Shanghai, China) were selected as representative lymphoma cell models to evaluate the proposed AI-assisted optical biosensing platform. Both cell lines were cultured in RPMI-1640 medium supplemented (Corning, NY, USA) with 10% fetal bovine serum, 100 U mL^−1^ penicillin, and 100 μg mL^−1^ streptomycin (Zhongqiaoxinzhou Biotech Co., Ltd., Shanghai, China) at 37 °C in a humidified incubator with 5% CO_2_. To minimize phenotypic variation, all experiments were performed using cells within 15 passages.

Prior to optical imaging, cells were washed twice with 0.01 M phosphate-buffered saline (PBS, Beyotime, Shanghai, China), fixed in 4% paraformaldehyde (Beyotime, Shanghai, China) at room temperature for 30 min to preserve cellular morphology, centrifuged at 1500 rpm for 3 min, and finally resuspended in PBS at a concentration of approximately 2.0 × 10^3^ cells mL^−1^ for subsequent microfluidic light-scattering imaging experiments.

### 2.2. Microfluidic Chip Fabrication

A polydimethylsiloxane (PDMS) based microfluidic chip was designed and fabricated to enable continuous-flow optical detection of individual cells. The PDMS prepolymer and curing agent (Sylgard 184, Dow Corning, Midland, MI, USA) were thoroughly mixed at a mass ratio of 10:1, and approximately 20 g of the mixture was poured into a customized mold. Stainless-steel capillary tubes with an outer diameter of 0.7 mm were embedded in the uncured PDMS to define both the microfluidic channels and the optical fiber insertion channel. The PDMS mixture was subsequently degassed under vacuum for approximately 1 h to remove trapped air bubbles and cured at 75 °C for 3 h.

After polymerization, the stainless-steel templates were carefully removed, and the cured PDMS slab was trimmed to approximately 24 mm × 60 mm to match the dimensions of the glass substrate. Transparent silicone tubing (0.3 mm inner diameter and 0.8 mm outer diameter) was connected to the inlet ports to provide fluidic access. The PDMS substrate and glass coverslip were treated with oxygen plasma and immediately bonded to form an irreversible seal. To further enhance the bonding strength and ensure stable operation during continuous-flow experiments, the assembled device was incubated at 75 °C for an additional 3 h.

The completed microfluidic chip consisted of a three-inlet Y-shaped channel configuration designed for hydrodynamic focusing. The central inlet was used for sample introduction, whereas the two lateral inlets delivered sheath fluid to confine cells into a narrow flow stream, thereby enabling stable single-cell interrogation within the optical detection region. In addition, a lateral channel positioned orthogonally to the microfluidic channel was incorporated to accommodate an optical fiber for excitation light delivery, facilitating reproducible acquisition of 2D light-scattering patterns under continuous-flow conditions. The area within 0.5 mm to the left and right near the optical fiber insertion port is defined as the optical interrogation range. The entire optical interrogation region is approximately a rectangle measuring 0.7 mm × 1 mm. The width of the focused light beam stays within the optical interrogation range, ensuring repeatable acquisition of 2D scattering patterns of single cells. This stable cell positioning method reduces fluctuations caused by deviations in cell motion trajectories and provides stable and reliable input data for subsequent deep learning-based cell classification tasks.

### 2.3. Microfluidic Light-Scattering Imaging Platform

**Figure 1:**
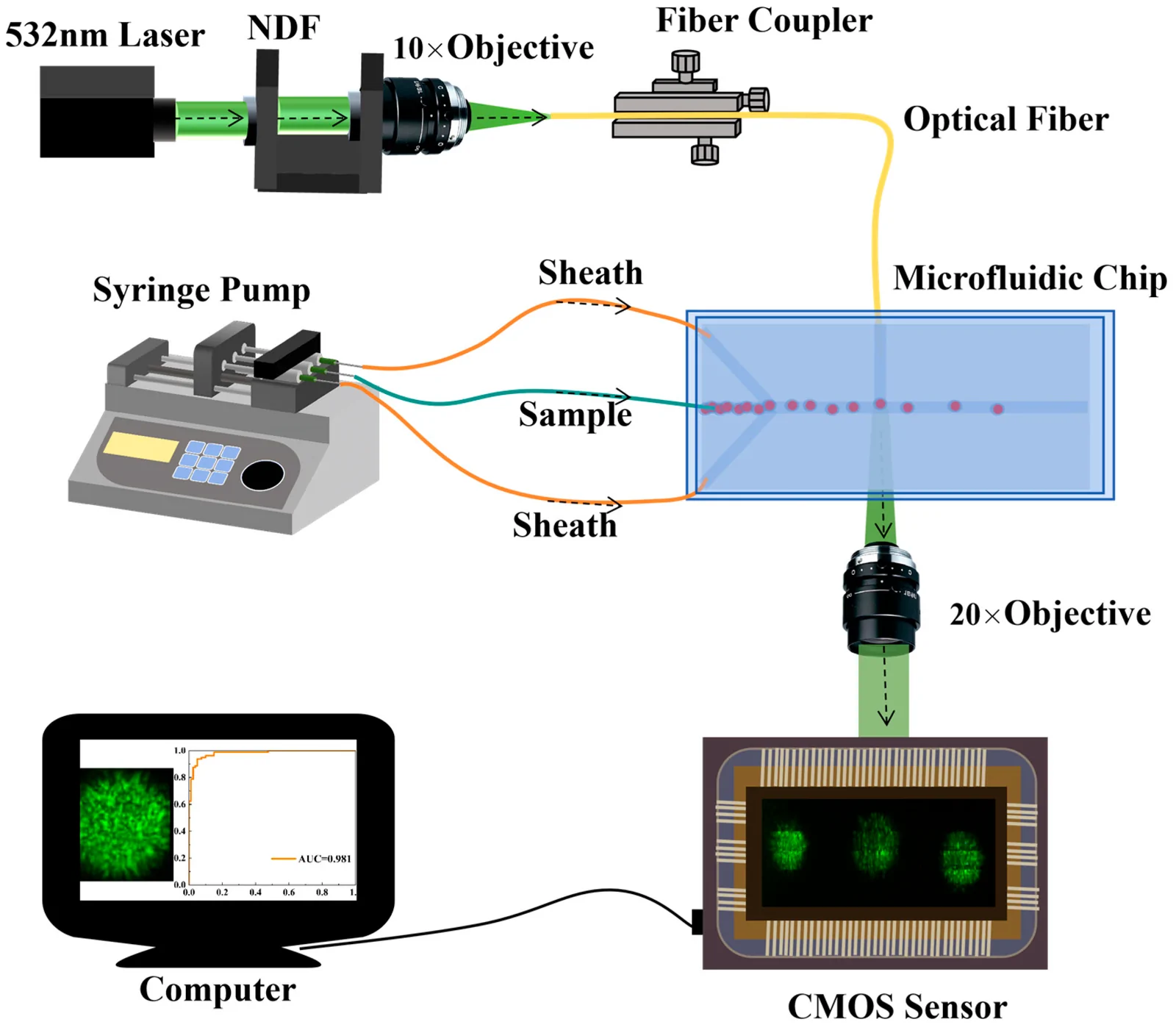
Schematic illustration of the AI-assisted microfluidic light-scattering imaging platform for label-free single-cell analysis. A 532 nm laser beam was coupled into a multimode optical fiber to illuminate microspheres or cells hydrodynamically focused within the microfluidic channel. The resulting 2D light-scattering patterns were collected by a 20× objective lens and recorded using a CMOS sensor for subsequent automated image processing and deep learning-based classification.

A microfluidic light-scattering imaging platform was developed for continuous-flow, label-free analysis of individual microspheres and lymphoma cells, as schematically illustrated in Figure 1. The platform integrates laser excitation, fiber-based light delivery, microfluidic hydrodynamic focusing, optical scattering imaging, and AI-assisted image analysis into a unified optical biosensing system for single-cell characterization.

A diode-pumped solid-state laser (DPSSL, Edmund Optics, Barrington, NJ, USA) operating at a wavelength of 532 nm with an output power of 50 mW served as the excitation source. To avoid excessive optical intensity at the detection region, the laser beam was attenuated using a neutral density filter (NDF) with a transmittance of 10% (Zolix, Beijing, China). The attenuated beam was focused through a 10× objective lens (LDA-Plan, Zeiss, Oberkochen, Germany) and coupled into a multimode optical fiber with a core diameter of 50 μm (Thorlabs, Newton, NJ, USA). The input end of the optical fiber was mounted on a three-axis translation stage (Zolix, China) to enable precise alignment and maximize coupling efficiency. The output end was inserted into the integrated optical fiber channel of the microfluidic chip, allowing excitation light to illuminate individual microspheres or cells passing through the optical interrogation region.

The microfluidic chip was mounted on the stage of an inverted optical microscope (Vert.A1, Zeiss, Germany). Cell suspensions were delivered by a syringe pump, while sheath flows confined the sample stream through hydrodynamic focusing, ensuring stable interrogation of individual particles or cells under continuous-flow conditions.

Scattered light generated by the interaction between the excitation beam and the flowing sample was collected using a 20× objective lens (LDA-Plan, Zeiss, Germany; numerical aperture, NA = 0.35) and recorded by a complementary metal-oxide-semiconductor (CMOS) sensor (Axiocam 503 color, Zeiss, Germany). The scattered light pattern was captured using a CMOS camera; the camera frame rate was set to 10 frames per second, with an exposure time of 50 ms. According to the optical detection geometry, the imaging system collected scattering signals over an angular range of approximately 75–105° in both the polar *θ* and azimuth φ directions (Document S1 [47,48]), enabling reproducible acquisition of 2D light-scattering patterns for subsequent image analysis and deep learning-based classification.

## 3. Results

### 3.1. Validation of the Microfluidic Light-Scattering Imaging Platform

**Figure 2:**
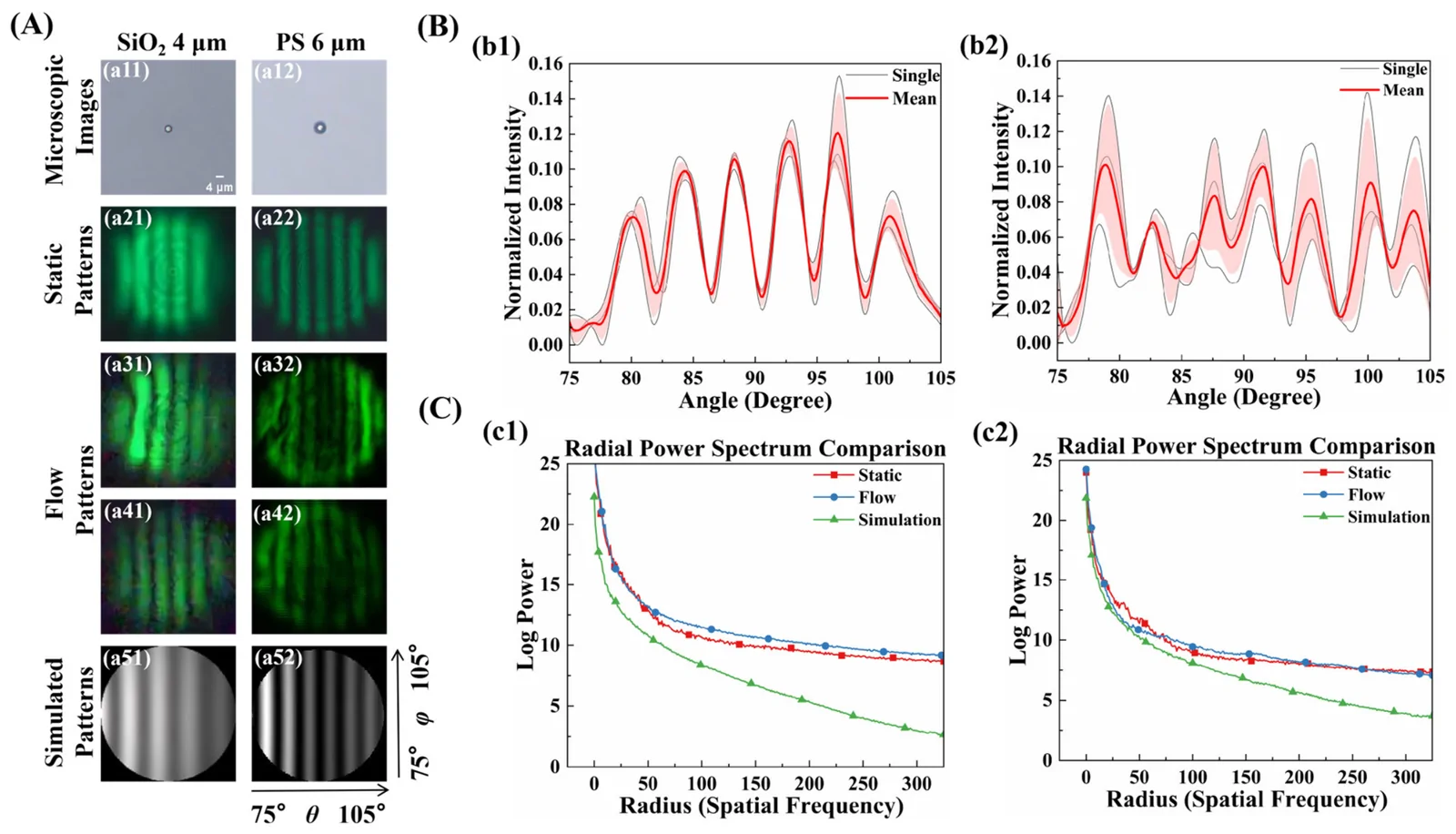
Experimental validation of the microfluidic light-scattering imaging platform. (**A**) Bright-field microscopic images, static light-scattering patterns, continuous-flow light-scattering patterns, and corresponding Mie theory-based simulated scattering patterns of 4 μm SiO_2_ and 6 μm PS microspheres. (**a11**) represents the SiO_2_ microspheres under bright-field microscopy, (**a21**) represents their static light-scattering pattern, (**a31**–**a41**) represent their flow light-scattering pattern, and (**a51**) represents their Mie simulation pattern. (**a12**) represents the PS microspheres under bright-field microscopy, (**a22**) represents their static light-scattering pattern, (**a32**–**a42**) represent their flow light-scattering pattern, and (**a52**) represents their Mie simulation pattern. (**B**) Angular intensity profiles extracted from the flow scattering patterns at φ = 90°. Gray curves represent individual measurements, red curves indicate the mean intensity profiles, and the shaded regions denote the corresponding standard deviations. (**b1**) represents the SiO_2_ microspheres, and (**b2**) represents the PS microspheres. (**C**) Comparison of radially averaged one-dimensional power spectral profiles derived from static, continuous-flow, and simulated scattering patterns following 2D FFT analysis. (**c1**) represents the SiO_2_ microspheres, and (**c2**) represents the PS microspheres.

To validate the optical performance of the proposed microfluidic light-scattering imaging platform prior to biological experiments, standard 4 μm SiO_2_ microspheres and 6 μm PS microspheres were employed as reference samples. Bright-field microscopic images, static light-scattering patterns, and flow light-scattering patterns were acquired under identical experimental conditions. Representative results are presented in Figure 2A, together with the corresponding scattering patterns simulated using Mie scattering theory. The theoretical patterns were generated in MATLAB (R2022a) using the measured particle diameters and refractive indices. For both microsphere types, the experimentally acquired scattering patterns exhibited characteristic fringe distributions and overall intensity profiles that closely agreed with the theoretical predictions, indicating that the proposed optical imaging platform accurately captured the intrinsic scattering characteristics of individual particles under both static and continuous-flow conditions.

To further evaluate the repeatability of dynamic light-scattering measurements, angular intensity profiles were extracted from the flow scattering patterns at an azimuthal angle of φ = 90°. For each microsphere type, three representative scattering patterns were analyzed individually, and the corresponding mean intensity profile was calculated. As shown in Figure 2B, the intensity profiles obtained from different microspheres exhibited highly consistent oscillatory features with only minor variations, while the shaded regions representing the standard deviation remained relatively narrow across the measured angular range. These results demonstrate the excellent repeatability and stability of the proposed imaging platform during continuous-flow optical measurements.

The accuracy of the acquired scattering patterns was further assessed in the spatial-frequency domain using 2D fast Fourier transform (FFT) analysis. Radially averaged one-dimensional power spectral profiles derived from the static, flow, and simulated scattering patterns are compared in Figure 2C. The spectra obtained from the static and flow measurements showed nearly identical distributions throughout the investigated spatial-frequency range, indicating that continuous-flow imaging preserved the intrinsic spatial-frequency characteristics of the scattering signals. Although the simulated spectra followed the same overall trend, their amplitudes were consistently lower than those of the experimental measurements. The discrepancy between the simulated and experimental radial power spectra is expected because the Mie model assumes ideal spherical particles with uniform refractive-index distributions, whereas experimental measurements are affected by several practical factors, including particle size distribution, optical aberrations, illumination nonuniformity, detector response, and background scattering.

Collectively, these results demonstrate that the proposed microfluidic optical biosensing platform enables accurate, stable, and reproducible acquisition of 2D light-scattering patterns under continuous-flow conditions. This robust optical measurement capability provides a reliable foundation for subsequent AI-assisted single-cell feature extraction and label-free lymphoma cell classification.

### 3.2. Dataset Acquisition and Construction

Following validation of the optical imaging platform, light-scattering data from Daudi and SUP-T1 lymphoma cells were acquired to establish a dataset for subsequent deep learning-based classification. Cell suspensions were introduced into the microfluidic chip at a flow rate of 500 μL h^−1^ using a syringe pump, while continuous videos of individual cells passing through the optical interrogation region were recorded. During actual experiments, due to the non-uniform distribution of cells in the solution and the fast flow of sheath fluid (3 mL h^−1^), the effective imaging rate is approximately 2 cells per second. Approximately 50 scattering videos, each with a duration of approximately 1 min, were collected for each lymphoma cell type under identical experimental conditions, providing sufficient raw data for automated single-cell extraction.

**Figure 3:**
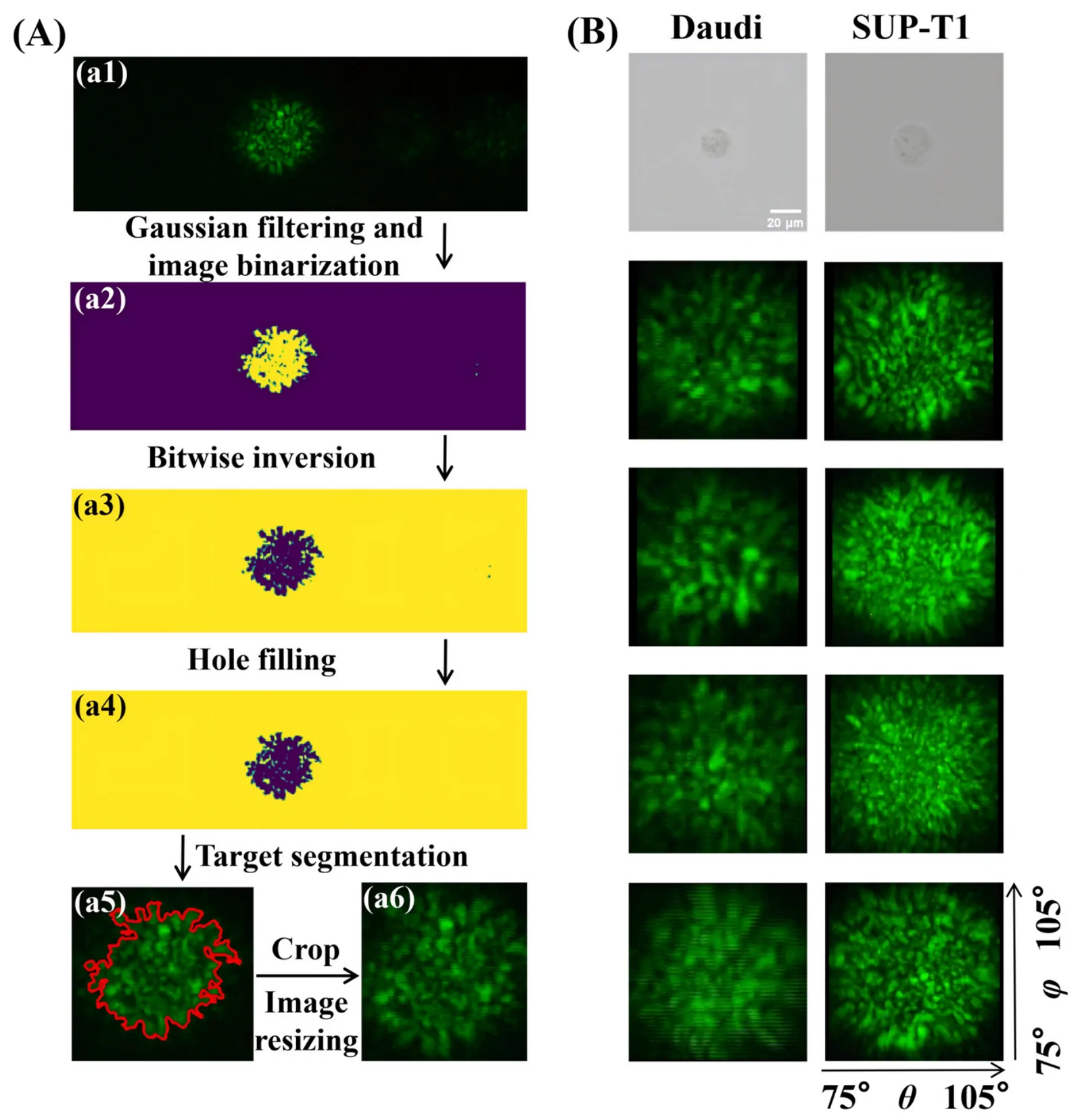
Automated dataset construction from continuous-flow light-scattering measurements. (**A**) Image-processing workflow for automatic extraction of individual cell light-scattering patterns from continuous-flow videos, including frame extraction, image binarization, Gaussian filtering, bitwise inversion, hole filling, target segmentation, cropping, and image resizing. (**a1**) is the video frame image. (**a2**) is the image after Gaussian denoising and binarization. (**a3**) is the image obtained by applying a bitwise inversion operation to the previous image. (**a4**) is the image obtained by hole filling on the basis of the previous image. (**a5**) is the image during the target extraction process. (**a6**) is the single-cell scattering image after target extraction. (**B**) Representative bright-field microscopic images and corresponding 2D light-scattering patterns of Daudi and SUP-T1 lymphoma cells following automated preprocessing.

To efficiently convert continuous-flow scattering videos into individual image samples, an automated image-processing pipeline was developed using Python (Python 3.9). As illustrated in Figure 3A, each video was sequentially decomposed into individual frames, followed by image binarization to simplify the image content and facilitate target identification. Gaussian filtering was subsequently applied to suppress background noise, after which bitwise inversion was performed to enhance the contrast between the scattering pattern and the background. Hole filling and target segmentation were then conducted to isolate individual scattering patterns from each frame. Finally, the extracted scattering patterns were cropped and resized to 224 × 224 pixels using a standardized preprocessing procedure to generate uniformly formatted input images for the deep learning model. The processing of these patterns is performed based on the video frames output by the original sensor, and no processing methods that may alter the spatial distribution of images are adopted.

To ensure the independence of the single-cell scattering pattern dataset, only one representative scattering pattern was selected from each individual cell trajectory. During continuous-flow video acquisition, a single cell may appear in multiple consecutive frames; therefore, these sequential frames were not treated as independent samples. Instead, after image segmentation and trajectory identification, the first complete 2D scattering pattern of each individual cell was selected for database construction. In addition, since the size of the microfluidic channel is larger than the cell diameter, misalignment may occur during cell flow. Therefore, juxtaposed cells are excluded when selecting single-cell scattering patterns. Following this selection criterion, 400 independent single-cell scattering patterns were obtained for each lymphoma cell type, resulting in a dataset containing 800 single-cell scattering patterns.

Representative bright-field microscopic images and the corresponding 2D light-scattering patterns of Daudi and SUP-T1 cells are presented in Figure 3B. Despite their similar morphology in bright-field images, the two lymphoma cell types exhibited distinct light-scattering distributions, suggesting that intrinsic optical scattering signatures may provide complementary information for cell discrimination. Following automated preprocessing, a total of 800 high-quality single-cell scattering images were obtained, comprising 400 Daudi and 400 SUP-T1 samples.

The automated processing pipeline substantially reduced manual intervention during dataset construction while ensuring consistent image quality and standardized preprocessing across all samples. This workflow enabled efficient transformation of continuous-flow optical measurements into a high-quality image dataset, providing a reliable basis for subsequent AI-assisted feature learning and label-free single-cell classification.

### 3.3. Deep Learning-Based Classification of Lymphoma Cells

To achieve automated label-free classification of lymphoma cells, this study adopts a transfer-learning strategy and uses the ImageNet-pretrained ResNet50 network for deep feature extraction. The original fully connected classification layers of ResNet50 were removed, and the convolutional backbone was used as a fixed feature extractor. Consequently, a 2048-dimensional feature representation is generated from each input scatter pattern. The extracted deep features were subsequently classified using a multilayer perceptron (MLP) classifier. The MLP consisted of two fully connected layers with 512 and 256 neurons, respectively. Rectified linear unit (ReLU) activation functions were applied after each dense layer, followed by dropout layers with dropout rates of 0.4 and 0.3 to reduce overfitting. The final output layer contained two neurons with softmax activation for binary lymphoma cell classification. The model was trained using the Adam optimizer with a learning rate of 1 × 10^−4^. The batch size and maximum training epochs were set to 24 and 100, respectively.

To evaluate the model performance, stratified five-fold cross-validation was performed on the single-cell image dataset. The dataset contained 400 Daudi and 400 SUP-T1 scattering patterns. In each fold, the dataset was randomly divided into training and validation subsets at a ratio of 8:2, while maintaining identical class distributions. Model performance was comprehensively evaluated using ROC analysis, confusion matrices, accuracy, precision, recall, F1-score, and area under the ROC curve (AUC).

**Figure 4:**
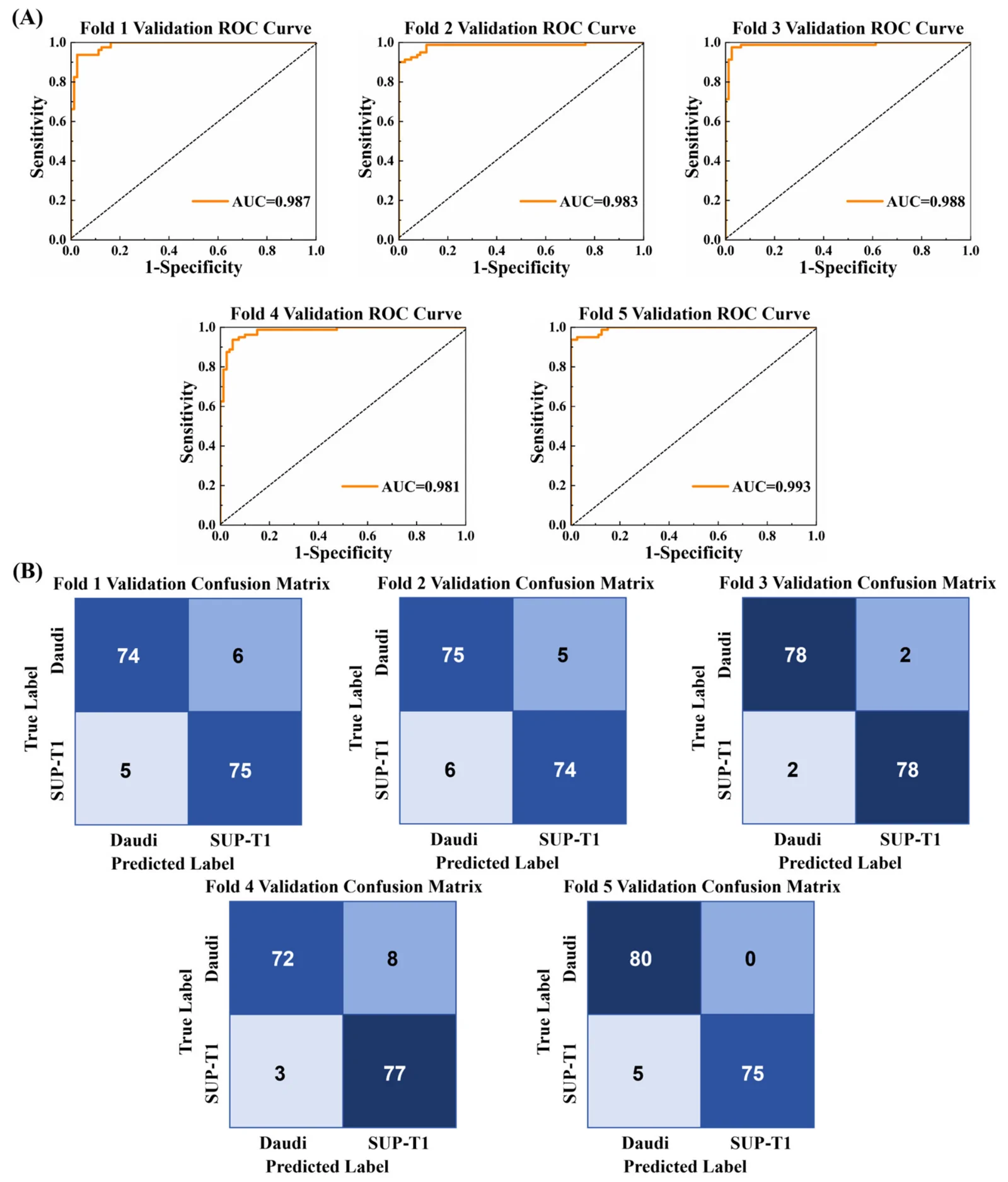
Performance evaluation of the ResNet50-based transfer learning model for label-free lymphoma cell classification. (**A**) The ROC curves obtained from stratified five-fold cross-validation. (**B**) Corresponding confusion matrices for each validation fold.

The ROC curves obtained from the five validation folds are presented in Figure 4A. All curves were concentrated near the upper-left corner of the ROC space, indicating excellent discrimination between the two lymphoma cell types. The corresponding AUC values ranged from 0.981 to 0.993, with a mean AUC of 0.986 (Table 1), demonstrating consistently high classification performance across different validation folds and confirming the robustness of the proposed framework.

The corresponding confusion matrices are shown in Figure 4B. For all validation folds, the majority of samples were correctly assigned to their respective classes, with only a small number of misclassifications. As summarized in Table 1, the proposed method achieved an average classification accuracy of 94.75%, reaching a maximum accuracy of 97.50% in Fold 3, while all remaining folds achieved accuracies above 93%. For Daudi cells, the average recall, precision, and F1-score were 94.75%, 94.78%, and 94.72%, respectively. The corresponding values for SUP-T1 cells were 94.75%, 94.87%, and 94.77%, respectively. The balanced performance across all evaluation metrics indicates that the proposed classifier effectively distinguished the two lymphoma cell types without introducing noticeable classification bias.

**Figure 5:**
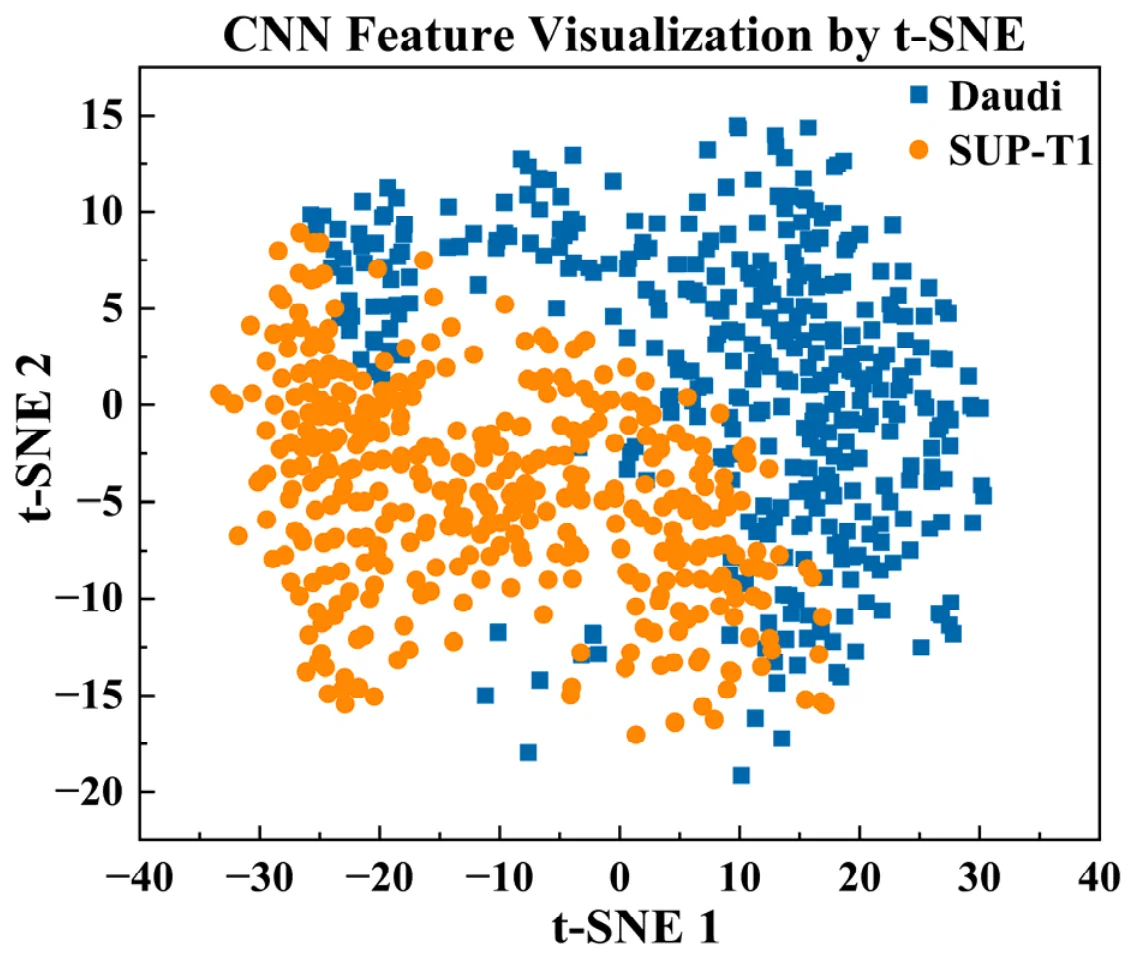
The t-SNE visualization of deep feature representations extracted by the pretrained ResNet50 network from 2D light-scattering images. Each point represents one single-cell scattering pattern, and different colors denote different lymphoma cell types.

To further investigate the discriminative capability of the learned deep representations, t-distributed stochastic neighbor embedding (t-SNE) was employed to visualize the high-dimensional feature space generated by the pretrained ResNet50 backbone. As shown in Figure 5, the extracted features formed two distinguishable clusters corresponding to Daudi and SUP-T1 cells. Although partial overlap was observed at the cluster boundaries, most samples were well separated in the embedded feature space, indicating that the network successfully learned discriminative optical scattering representations associated with different lymphoma cell types. Although t-SNE visualization provides intuitive insight into the distribution of learned features, it interpreted only as qualitative evidence. The actual classification capability of the proposed model was determined based on quantitative evaluation metrics.

Collectively, these results demonstrate that the proposed AI-assisted optical biosensing framework effectively combines continuous-flow microfluidic light-scattering imaging with transfer learning to achieve accurate label-free single-cell classification. The high classification accuracy, robust cross-validation performance, and clear feature separability indicate that 2D light-scattering patterns contain sufficient intrinsic optical information for reliable discrimination of lymphoma cell types without fluorescent labeling.

## 4. Discussion

The present study demonstrates that integrating microfluidic light-scattering imaging with deep learning provides an effective strategy for label-free single-cell classification of lymphoma cells. By combining continuous-flow optical detection with transfer learning, the proposed platform successfully transformed 2D light-scattering patterns into highly discriminative optical representations, enabling accurate identification of Daudi and SUP-T1 lymphoma cells without fluorescent labeling or molecular staining. The high classification accuracy (94.75%) and consistently high AUC values obtained from five-fold cross-validation further demonstrate the robustness and reliability of the proposed AI-assisted optical biosensing framework.

A key advantage of the proposed platform lies in its combination of microfluidics and optical scattering imaging. Hydrodynamic focusing within the microfluidic chip enabled stable interrogation of individual cells under continuous-flow conditions, while fiber-coupled laser excitation provided reproducible acquisition of high-quality scattering patterns. These results indicate that the developed platform provides reliable optical measurements suitable for subsequent AI-based feature extraction and classification.

Compared with conventional machine learning approaches for optical scattering analysis, the proposed transfer learning framework substantially simplifies the analytical workflow. Traditional classification methods generally rely on handcrafted feature extraction, requiring extensive prior knowledge and manual feature engineering to characterize scattering images. In contrast, the pretrained ResNet50 backbone automatically learns hierarchical feature representations directly from 2D scattering patterns, allowing discriminative optical information to be extracted in an end-to-end manner. This data-driven strategy not only improves classification performance but also enhances model adaptability for future applications involving different cell types or optical imaging conditions.

From the perspective of optical biosensing, the proposed framework demonstrates the potential of combining artificial intelligence with label-free optical imaging for intelligent cellular analysis. Unlike fluorescence-based assays that require labeling procedures and may alter cellular physiology, this platform does not require fluorescent antibodies or molecular labeling, and can directly analyze cell scattering properties related to cell morphology, intracellular structures and refractive-index distribution. The built optical system does not require repeated optical alignment and adjustment after initial commissioning. These components enable controlled single-cell interrogation and optical signal acquisition under continuous-flow conditions. The major advantage of this platform is not superior analytical capacity compared with commercial flow cytometry, but its capability for label-free optical phenotypic analysis, which focuses on the rapid acquisition and computational interpretation of intrinsic optical characteristics of cells. Integrating these label-free optical measurements with deep learning enables automatic interpretation of complex scattering information, providing a promising solution for rapid and high-throughput single-cell analysis. The current system is particularly suitable for preliminary screening, research-oriented cellular phenotyping, and applications where preservation of intrinsic cellular properties is desirable. Future integration with additional optical or biochemical sensing modalities, together with larger clinical datasets, may further expand its analytical capability and translational potential. Such a strategy is well aligned with the emerging trend of AI-enabled optical biosensors for intelligent healthcare applications.

Several limitations should nevertheless be acknowledged. First, the current study represents a proof-of-concept validation using two established lymphoma cell lines (Daudi and SUP-T1) under controlled laboratory conditions. Although this confirms the feasibility of distinguishing lymphoma cells based on intrinsic light scattering characteristics, they cannot fully reflect the biological heterogeneity encountered in clinical lymphoma diagnosis. Therefore, in future studies, we plan to expand this classification framework to cover a broader spectrum of lymphoma subtypes. By further optimizing the optical system configuration and data-processing workflow, we aim to achieve robust discrimination among multiple lymphoid cell types, followed by clinical validation using patient-derived samples to rigorously evaluate the diagnostic potential of this system. Second, only 2D light-scattering patterns were used for classification. Incorporating complementary sensing modalities, such as Raman spectroscopy, fluorescence imaging, or electrical impedance measurements, may further improve classification accuracy by providing multimodal biochemical and structural information. Third, although transfer learning based on ResNet50 achieved excellent performance, recently developed deep learning architectures, including vision transformers and multimodal fusion networks, may provide stronger feature learning capability and should be explored in future investigations.

Overall, this study demonstrates that the synergistic integration of microfluidics, optical scattering imaging, and artificial intelligence constitutes a promising framework for label-free optical biosensing. Beyond lymphoma cell classification, the proposed strategy may be extended to other biomedical applications requiring rapid, automated, and high-throughput single-cell analysis, including hematological disease screening, circulating tumor cell detection, and intelligent cellular phenotyping.

## 5. Conclusions

In this study, we developed an AI-assisted microfluidic light-scattering imaging platform for label-free single-cell classification of lymphoma cells. The proposed system integrates continuous-flow microfluidic manipulation, fiber-based optical excitation, 2D light-scattering imaging, automated image preprocessing, and transfer learning based on a pretrained ResNet50 network into a unified optical biosensing framework. Experimental validation demonstrated that the platform enables stable acquisition of high-quality light-scattering patterns under continuous-flow conditions and achieves accurate classification of Daudi and SUP-T1 lymphoma cells with an average accuracy of 94.75% and an average AUC of 0.986.

The proposed framework eliminates the need for fluorescent labeling while maintaining high classification performance, highlighting the capability of artificial intelligence to enhance the analytical power of optical biosensors. Owing to its simplicity, automation, and compatibility with continuous-flow detection, this platform represents a promising approach for intelligent single-cell analysis. Upon further validation using multiple lymphoma subtypes and clinical samples, the platform can provide complementary optical information for future applications in single-cell screening and phenotypic characterization. It holds broad application prospects in future fields including clinical screening, disease diagnosis, disease monitoring, and precision medicine.

## Figures and Tables

**Table 1:** Classification performance of the transfer learning model based on 2D light-scattering features obtained from stratified five-fold cross-validation.

	Cell Types	Cross-Validation Folds					Mean ± 95% CI
		Fold 1	Fold 2	Fold 3	Fold 4	Fold 5	
Recall	Daudi	92.5%	93.75%	97.5%	90%	100%	94.75% ± 4.96%
	SUP-T1	93.75%	92.5%	97.5%	96.25%	93.75%	94.75% ± 2.55%
Precision	Daudi	93.67%	92.59%	97.5%	96%	94.12%	94.78% ± 2.43%
	SUP-T1	92.59%	93.67%	97.5%	90.59%	100%	94.87% ± 4.74%
F1-Score	Daudi	93.08%	93.17%	97.5%	92.9%	96.97%	94.72% ± 2.86%
	SUP-T1	93.17%	93.08%	97.5%	93.33%	96.77%	94.77% ± 2.70%
AUC		0.987	0.983	0.988	0.981	0.993	0.986 ± 0.006
Accuracy		93.13%	93.13%	97.5%	93.13%	96.88%	94.75% ± 2.77%

## Data Availability

Data underlying the results presented in this paper are not publicly available at this time but may be obtained from the authors upon reasonable request.

## Supplementary Materials

The following supporting information can be downloaded at https://www.mdpi.com/article/10.3390/bios16090500/s1, Document S1: Calculation of scattering angle, Figure S1: Schematic diagram of particle scattering; Figure S2: Schematic diagram of scattered light from laser-excited standard microsphere; Figure S3: Schematic diagram of intersection between scattered light at different polar angles and the photosensitive surface of CMOS sensor; Figure S4: Schematic diagram of the azimuth angle of scattered light detected by the CMOS sensor; Figure S5: Light path schematic diagram for the excitation particles.

## Abbreviations

The following abbreviations are used in this manuscript:

2D	Two-dimensional
ROC	Receiver operating characteristic
SiO_2_	Silica
PS	Polystyrene
PBS	Phosphate-buffered saline
PDMS	Polydimethylsiloxane
DPSSL	Diode-pumped solid-state laser
NDF	Neutral density filter
CMOS	Complementary metal-oxide-semiconductor
FFT	Fast Fourier transform
MLP	Multilayer perceptron
ReLU	Rectified linear unit
AUC	Area under the ROC curve
t-SNE	t-distributed stochastic neighbor embedding
