# Supplementary material for: Microfluidic Light-Scattering Imaging Coupled with Deep Learning for Label-Free Single-Cell Classification of Lymphoma Cells
Source: Biosensors (Basel). 2026 Sep 7;16(9):500. doi: 10.3390/bios16090500 (PMC13604312; doi:10.3390/bios16090500)
Supplement: Supplementary file 1 [file biosensors-16-00500-s001.zip › biosensors-4506769-supplementary.pdf]

Supplementary Materials

Microfluidic Light-Scattering Imaging Coupled with Deep Learning for Label-Free Single-Cell Classification of Lymphoma Cells

Calculation of Scattering Angle

In 1908, German physicist Gustav Mie obtained the rigorous solution for elastic scattering by a homogeneous dielectric sphere by solving the boundary conditions of Maxwell's equations [1]. This solution enables the calculation of scattered light from spherical particles with different sizes and refractive indices, which is known as the Mie scattering theory. The scattering process of spherical particles is shown in Figure S1.

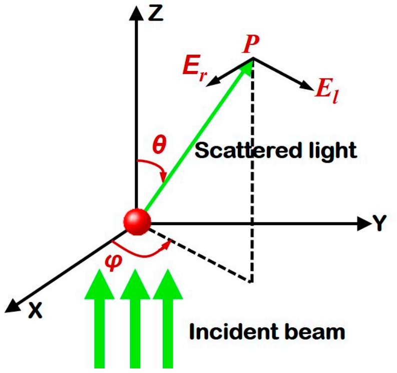

Figure S1. Schematic diagram of particle scattering.

The scattering particles are located at the coordinate origin. The incident light enters along the positive direction of the Z-axis, and the direction of the electric field vibration is parallel to the X-axis. *d* is the diameter of the spherical particles, λ is the wavelength of the incident light, r is the distance between the center point of the scattering particles and the observation point P, and the angle between the Z-axis and the scattering direction is the polar angle θ, and the angle between the x-axis and the projection of the scattered light on the XY plane is azimuth angle φ, the scattered light intensity is:

$$\mathrm{I}(r,\theta,\varphi)=\frac{\lambda^2 I_0}{4\pi^2 r^2}\left[i_1(\theta)\sin^2\varphi+i_2(\theta)\cos^2\varphi\right]$$

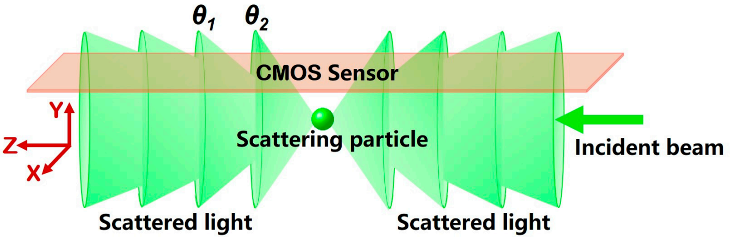

Figure S2. Schematic diagram of scattered light from laser-excited standard microsphere.

Based on Mie scattering theory, when a homogeneous standard microsphere is vertically illuminated by a laser beam, a series of conical scattering waves with different apex angles are generated [2], as shown in Figure S2. Different apex angles correspond to different polar angles, θ. Scattered light within a certain angular range passes through the microscope objective and intersects the photosensitive surface of the CMOS sensor, forming a series of parabolic curves. Because the center of the detection region is located on the optical axis of the microscope objective and is approximately symmetric about this axis, the CMOS sensor detects a series of hyperbolic curves with different curvatures that are approximately symmetric with respect to the optical axis of the objective. Figure S3 illustrates the intersections between the scattered-light cones corresponding to polar angles θ_1 and θ_2 and the CMOS sensor.

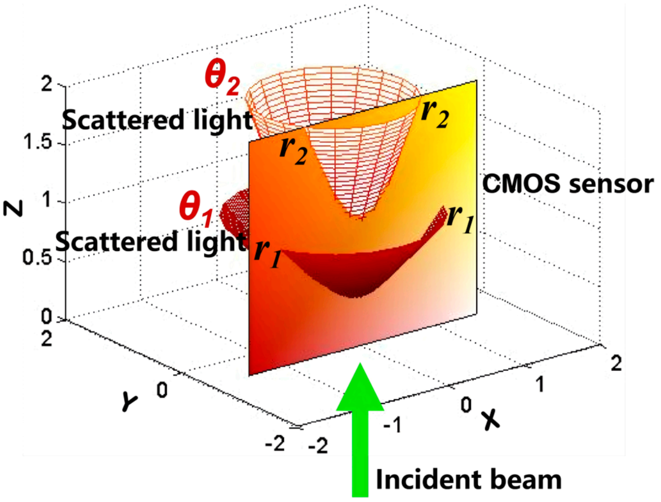

Figure S3. Schematic diagram of intersection between scattered light at different polar angles and the photosensitive surface of CMOS sensor.

The plane containing the scattered light corresponding to $\theta = 90°$ is defined as the XY plane. For convenience, only the case of $Z \geq 0$ is considered in Figure S3. The Z axis represents the propagation direction of the incident light emitted from the fiber-optic probe, while the X axis is aligned with the electric-field vector of the incident light. The points r_1 and r_2 are observation points, representing the endpoints of the intersection curves between the CMOS detector and the scattering cones with apex angles θ_1 and θ_2, respectively. The plane defined by an observation point and the Z axis is referred to as the scattering plane, and the angle between the electric-field vector of the incident light and the scattering plane is defined as the azimuth angle φ, as illustrated schematically in Figure S4. When the vertex angle, namely the polar angle, is θ_1, the corresponding azimuth angle range is given by: $\varphi_1 \leq \varphi \leq \varphi_2$. The projection of the intersection curve between the scattering cone with polar angle θ and the CMOS detector onto the XY plane is a line segment, whose length is denoted by d. For scattering light generated by the same particle, a larger value of d indicates a broader range of azimuth angles corresponding to the given polar angle θ. As shown in Figure S4, the projected line segments corresponding to polar angles θ_1 and θ_2 have lengths d_1 and d_2, respectively. Since $d_1 \geq d_2$, the azimuth angle range corresponding to θ_1 is greater than that corresponding to θ_2. Notably, when $\theta = 90°$, the corresponding azimuth angle range reaches its maximum, which is consistent with the maximum polar angle range that can be detected by the CMOS detector.

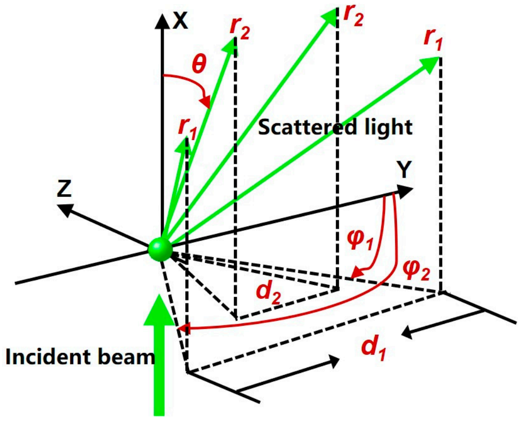

Figure S4. Schematic diagram of the azimuth angle of scattered light detected by the CMOS sensor.

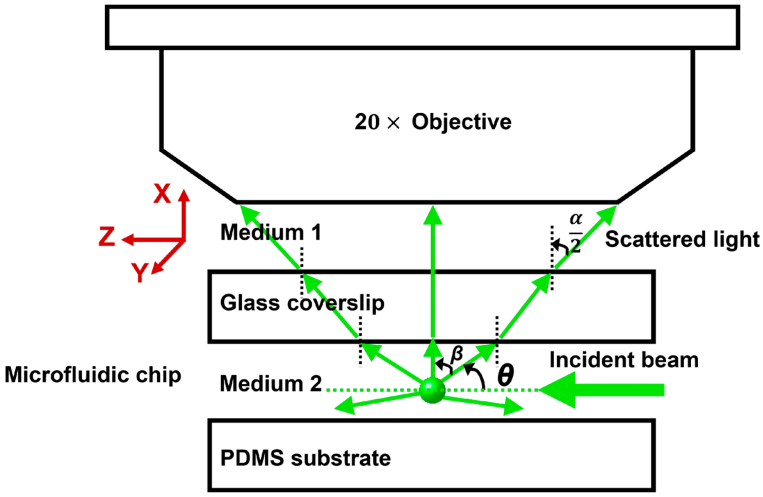

Figure S5. Light path schematic diagram for the excitation particles.

As shown in Figure S5, the scattered light from the laser-excited particles propagates in Medium 2 (refractive index n_2), the medium where the particles are suspended, to the lower surface of the Glass coverslip. It is refracted into the Glass coverslip, and then refracted into Medium 1 (refractive index n_3), the medium connecting the microfluidic chip and the front end of the microscope objective. The scattered light enters the microscope objective from Medium 1. The numerical aperture (NA) is an important parameter of a microscope objective and determines its light-gathering capability and spatial resolution. In the present study, the NA also determines the angular range of scattered light that can be detected by the microfluidic light-scattering imaging platform. The numerical aperture (NA), also referred to as the aperture ratio, is defined by the following equation:

$$\mathrm{NA} = n_3 * \sin\left(\frac{\alpha}{2}\right) \tag{1-1}$$

Where n_3 is the refractive index of Medium 1 between the front end of the microscope objective and the microfluidic chip; α is the aperture angle of the objective lens. The larger the aperture angle, the more scattered light enters the objective lens.According to Eq. (1-1):

$$\frac{\alpha}{2} = \mathrm{arc}\sin\left(\frac{NA}{n_3}\right) \tag{1-2}$$

It can be inferred from the law of refraction described by Snell concerning the relationship among the angle of incidence, the angle of refraction and the refractive index of the medium：

$$n_3 * \sin\left(\frac{\alpha}{2}\right) = n_2 * \sin\beta \tag{1-3}$$

Where n_2 is the refractive index of the medium surrounding the scattering particle, i.e., the refractive index of Medium 2, and β is the angle between the outermost scattered ray emitted by the scattering particle that can enter the microscope objective and the optical axis.

The angle between the excitation beam emitted by the laser and the scattered ray is defined as the polar angle θ, whereas the angle between the scattering plane and the polarization plane of the incident laser beam is defined as the azimuth angle φ. Accordingly, the detectable ranges of the polar angle θ and azimuthal angle φ can be determined as follows:

$$\left\{\begin{matrix} 90° - \beta \le \theta \le 90° + \beta \\ 90° - \beta \le \varphi \le 90° + \beta \end{matrix}\right\} \tag{1-4}$$

In this study, a 20× objective lens with NA = 0.35 was used. The refractive index of Medium 2 is n_2=1.334, and β can be calculated from Equations (1-1)-(1-3) as:

$$\beta = \arcsin\left(\frac{NA}{n_2}\right) = \arcsin\left(\frac{0.35}{1.334}\right) \approx 15.2°$$

From Equation (1-4), the ranges of the detectable polar angle θ and azimuth angle φ in this study can be calculated as:

$$90° - \beta \le \theta \le 90° + \beta \longrightarrow 74.8° \le \theta \le 105.2°$$
$$90° - \beta \le \varphi \le 90° + \beta \longrightarrow 74.8° \le \varphi \le 105.2°$$

which is approximated as 75°–105° in the manuscript.

References

1. Mie, G. Beiträge zur Optik trüber Medien, speziell kolloidaler Metallösungen. *Ann. Phys.* **1908**, *330*, 377–445.
2. Linyan Xie. Study of two-dimensional light scattering static cytometry and its application for leukemia detection[D]. Shandong University, 2018.
